# Characterization of disease course and remission in early seropositive rheumatoid arthritis: results from the TACERA longitudinal cohort study

**DOI:** 10.1177/1759720X211043977

**Published:** 2021-10-21

**Authors:** RA-MAP Consortium, Michael Barnes

**Keywords:** disease activity, latent class–mixed models, remission, rheumatoid arthritis, SDAI trajectories

## Abstract

**Background:**

To characterise disease course and remission in a longitudinal observational study of newly diagnosed, initially treatment-naïve patients with seropositive rheumatoid arthritis (RA).

**Methods:**

Patients with early untreated seropositive RA were recruited from 28 UK centres. Multiple clinical and laboratory measures were collected every 3 months for up to 18 months. Disease activity was measured using the 28-joint Disease Activity Score with C-reactive protein (DAS28-CRP) and Simplified Disease Activity Index (SDAI). Logistic regression models examined clinical predictors of 6-month remission and latent class mixed models characterised disease course.

**Results:**

We enrolled 275 patients of whom 267 met full eligibility and provided baseline data. According to SDAI definition, 24.3% attained 6-month remission. Lower baseline Health Assessment Questionnaire (HAQ) and SDAI predicted 6-month remission (*p* = 0.013 and 0.011). Alcohol intake and baseline prescribing of methotrexate with a second disease-modifying antirheumatic drug (DMARD; vs monotherapy without glucocorticoids) were also predictive. Three distinct SDAI trajectory subpopulations emerged; corresponding to an inadequate responder group (6.5%), and higher and lower baseline activity responder groups (22.4% and 71.1%). Baseline HAQ and Short Form-36 Health Survey – Mental Component Score (SF-36 MCS) distinguished these groups. In addition, a number of baseline clinical predictors correlated with disease activity severity within subpopulations. Beneficial effects of alcohol intake were found across subpopulations.

**Conclusion:**

Three distinct disease trajectory subpopulations were identified. Differential effects of functional and mental well-being, alcohol consumption, and baseline RA medication prescribing on disease activity severity were found across subpopulations. Heterogeneity across trajectories cannot be fully explained by baseline clinical predictors. We hypothesise that biological markers collected early in disease course (within 6 months) may help patient management and better targeting of existing and novel therapies.

## Introduction

Early diagnosis and prompt, tight control of disease activity have been proven to alter long-term course of rheumatoid arthritis (RA) and limit structural damage and long-term disability.^1^ In spite of a range of synthetic and biological disease-modifying antirheumatic drugs (DMARDs), clinical remission is achieved and sustained in a minority, and drug-free remission remains rare.^2^ This may be due to many factors. For example, there are no validated instruments for reliably predicting prognosis. Nor is it possible to confidently predict which patients will respond more favourably to one particular drug or drug combination over another. Furthermore, our understanding of low disease activity states is limited.^3,4^ The ability to employ the most appropriate ‘treat-to-target strategy’ to the right set of early RA patients would be a major advance. Recent evidence suggests that for early RA patients following a treat-to-target strategy, distinct trajectories of disease activity over the first year exist.^5^ Moreover, evidence from long-term observational cohorts has suggested that distinct trajectories linked to prognosis exist; which may point to distinct immunopathogenic subsets.^6^ Even anticitrullinated protein antibody (ACPA)-positive disease is heterogeneous in outcomes (e.g. radiological progression), further adding to arguments favouring a stratified medicine approach.^7,8^

To facilitate the goals of the RA MRC/ABPI (RA-MAP) Consortium,^9^ a cohort of newly diagnosed seropositive RA patients – the ‘Towards A CurE for RA’ (TACERA) cohort – was established and followed frequently and deeply phenotyped. The aim of this study was two-fold. First, we aimed to determine if baseline clinical factors and RA-prescribed medications are associated with 6-month remission in seropositive early RA. The 6-month time point was chosen to evaluate the primary outcome of remission as this corresponds to when therapy escalation would generally be considered following NICE guidelines. However, an analysis at a single time point neither reflects longitudinal disease activity patterns nor what factors predict, for example, sustained remission, fluctuating disease activity course, or gradual *versus* rapid response. Therefore second, we aimed to (1) determine whether different types of disease activity trajectory subpopulations exist within this inception cohort; (2) identify factors associated with longitudinal disease activity; and (3) determine whether differences in trajectory types (if they exist) associate with disease outcomes, baseline RA-prescribed medication, smoking status, and alcohol consumption. Identification of factors associated with disease activity and characterization of subgroups may improve patients’ treatment and management and identify the most suitable patients for recruitment into trials.

## Methods

### Patients

We recruited newly diagnosed patients ⩾18 years of age with symptom duration <12 months who fulfilled either the 1987 American College of Rheumatology (ACR) or 2010 European League Against Rheumatism (EULAR)/ACR classification criteria for RA.^10,11^ Patients were required to be ACPA and/or rheumatoid factor (RF) positive and naïve to DMARDs or glucocorticoid therapy. In addition, for patients recruited, there needed to be a clear intention by the supervising rheumatologist to commence therapy with DMARDs. Patients were excluded if they had significant comorbidities or if pregnant or wishing to conceive. Participation in trials impacting on patient’s treatment, immune status, or disease activity during the study period was not permitted. Ethical approval was authorised by the National Research Ethics Service London Central Committee (Reference number: 12/LO/0469). Informed, written consent was obtained.

### Study design

Subjects were recruited from 28 UK centres. Study sample size was determined as described in the Supplementary Material. Following enrolment, subjects received treatment using conventional synthetic DMARDs (csDMARDs), with therapy adjustments made at the supervising rheumatologist’s discretion according to the National Institute for Health and Clinical Excellence (NICE) guidelines for RA management in adults.^12^ Patients were followed for a period of 18, 12, or 6 months, dependent on time of enrolment (i.e. pre 01/07/14, 01/07/14-31/12/14 and post 31/12/14), and seen quarterly for scheduled assessments. Clinical, laboratory, lifestyle, medication, patient-reported outcome measures (PROMs), extra-articular RA features, adverse events, and biological samples were collected at visits. In addition, radiographs of the hands and feet were performed at baseline and 12 months or baseline and 6 months for subjects entering within the third enrolment period.

### Biological treatment

Subjects with inadequate clinical responses to csDMARDs and persistent high disease activity (DAS28 > 5.1) at 6 months could receive biological DMARDs (bDMARDs), according to NICE guidelines.

### Outcome measures

The co-primary outcomes were disease remission at 6 months and repeated disease activity over time. Disease activity was measured using both composite 28-joint Disease Activity Score with C-reactive protein, DAS28-CRP (4-component), and Simplified Disease Activity Index (SDAI) scores.^13–16^ Disease remission was defined using both DAS28-CRP remission criterion (DAS28-CRP < 2.6) and the more stringent SDAI criterion (SDAI ⩽ 3.3).

Secondary outcome measures included the ACR/EULAR Boolean definition of remission,^17^ 28-joint Disease Activity Score with erythrocyte sedimentation rate (DAS28-ESR), annualised rate of radiographic damage progression as measured by modified Larsen’s score,^18–20^ functional disability as measured by the Health Assessment Questionnaire (HAQ),^21^ and quality of life using both the Medical Outcomes Study Short Form-36 Health Survey (SF-36) and EuroQoL five dimensions questionnaire (EQ-5D).^22–24^

### Statistical methods

Baseline and 6-month information were summarised using the mean, with accompanying standard deviation (SD) for continuous variables, while binary or categorical variables were summarised using frequency and percentage.

To identify baseline predictors of 6-month clinical remission, a two-stage approach was adopted. In the first stage, variables, previously identified from the RA literature^25–30^ as potential predictors of remission, were univariately screened (using univariate logistic regression models with a conservative screen positive *p* value threshold of *p* < 0.2). Those screened positive in the first stage were taken forward to the second stage and included in multivariate logistic regression models that additionally included baseline prescribing of (or baseline intention to prescribe and then administered within 3 months) RA medication. Considered predictors in the first-stage comprised age, sex, ethnicity, body mass index (and obesity), symptom duration, smoking status, baseline disease activity, HAQ, SF-36 Mental Component Score (SF-36 MCS), alcohol consumption, serology (RF and ACPA), smoking, and erosion.

Latent class mixed models (LCMMs) were used to cluster disease activity trajectories that may identify clinically important subpopulations and characterise disease activity longitudinally. Within each latent class, fixed and random patient-level intercepts and piecewise linear time effects (at 5 months) were considered for the linear mixed-model part along with potential predictors informed from the earlier analyses on clinical remission. No covariates were included in the class-membership model part. LCMMs are likelihood-based methods, which are valid using only observed data, under a missing-at-random assumption. Associations of latent trajectory classes with outcomes, baseline RA medication prescribing, alcohol and smoking status were assessed either using analysis of variance or Fisher’s exact test. For the purpose of these association tests and more generally, patients were hard assigned to a particular latent trajectory class based on a posterior classification of class membership through the selection of the patient’s class with the highest estimated posterior class-membership probability. The best choice of the number of latent classes (3 classes vs 4 classes) was made using Bayesian Information Criterion (BIC) and relative entropy.

Statistical analyses were performed using the base library glm function for logistic regression and the hlme function in the lcmm package in the *R* statistical software.^31^ Two patients, not prescribed RA medications within the first 3 months, were excluded from the regression analyses.

The reporting of this study conforms to the Strobe statement.^32^

## Results

### Patient characteristics

Two hundred and seventy-five patients were recruited of whom 270 fulfilled all eligibility criteria. Two eligible patients withdrew at baseline without providing any clinical information. A further patient who withdrew at baseline had some clinical information but insufficient to calculate disease activity scores. Table 1 summarises the baseline characteristics of the remaining 267 patients. Briefly, the mean age at entry was 53.1 (SD of 15.2); 72% were female, 72.7% white, 31.8% healthy weight and the mean Charlson’s Comorbidity Index (CCI),^33^ modified to exclude rheumatic disease as this applied to all subjects, was 0.44 (SD of 0.84), conforming to the intention of not recruiting patients with significant comorbidities.

**Table 1.** Baseline characteristics of patients in the TACERA study.

Characteristics	Baseline (*n* = 267)	Missing data frequency
Age, years	53.1 (15.2)	0
Female	192 (71.9%)	0
White ethnicity	194 (72.7%)	0
BMI		0
Female	27.53 (6.47)	
Male	27.43 (4.97)	
Overall	27.50 (6.08)	
BMI status		0
Underweight: <18.5	9 (3.4%)	
Healthy weight: (18.5,25)	85 (31.8%)	
Overweight: (25,30)	95 (35.6%)	
Obese: ⩾30	78 (29.2%)	
Smoking		0
Never smoked	95 (35.6%)	
Previous smoker	104 (39.0%)	
Current smoker	68 (25.4%)	
Alcohol consumption		1
None	86 (32.3%)	
1–5 units per week	115 (43.2%)	
6–10 units per week	25 (9.4%)	
11–15 units per week	11 (4.1%)	
16–20 units per week	15 (5.7%)	
More than 20 units per week	14 (5.3%)	
Alcohol frequency		0
Not drinking	86 (32.2%)	
1–2 days a year	28 (10.5%)	
1–2 days a month	48 (18.0%)	
1–2 days a week	58 (21.7%)	
3–4 days a week	28 (10.5%)	
5 days or more a week	19 (7.1%)	
RF positive	247 (92.5%)	0
ACPA positive	230 (86.1%)	0
Disease duration (years)	0.43 (0.23)	0
X-ray Larsen’s Score (hands and feet)	6.70 (8.76)	6
Charlton’s Comorbidity Index (original)	0.44 (0.84)	0
Charlton’s Comorbidity Index (2008)^34^	0.81 (1.10)	0
SDAI	28.80 (14.29)	3
DAS28-CRP	4.85 (1.22)	2
Prescribed medication		0
Methotrexate (MTX)	202 (75.7%)	
Hydroxychloroquine	141 (52.8%)	
Leflunomide	0 (0.0%)	
Sulfasalazine	18 (6.7%)	
Oral glucocorticoids	17 (6.4%)	
Parenteral glucocorticoids	126 (47.2%)	
No RA medication	2 (0.7%)	
Medication combinations prescribed		0
No RA medication	2 (0.7%)	
MTX only	51 (19.1%)	
Other DMARDs only	20 (7.5%)	
Oral glucocorticoids only	2 (0.7%)	
Parenteral glucocorticoids only	15 (5.6%)	
MTX and other DMARDs	53 (19.9%)	
MTX and oral glucocorticoids	6 (2.2%)	
MTX and parenteral glucocorticoids	33 (12.4%)	
Other DMARDs and oral glucocorticoids	2 (0.7%)	
Other DMARDs and parenteral glucocorticoids	23 (8.6%)	
Oral and parenteral glucocorticoids	1 (0.4%)	
MTX, other DMARDs and oral glucocorticoids	5 (1.9%)	
MTX, other DMARDs and parenteral glucocorticoids	53 (19.9%)	
MTX, oral and parenteral glucocorticoids	1 (0.4%)	
Medication pyramid		0
No RA medication	2 (0.7%)	
MTX only	51 (19.1%)	
Other DMARDs without glucocorticoids	73 (27.4%)	
Glucocorticoids with/without other RA medication	141 (52.8%)	

ACPA, anticitrullinated protein antibody; BMI, body mass index; DAS28-CRP, 28-joint Disease Activity Score with C-reactive protein; MTX, methotrexate; RA, rheumatoid arthritis; RF, rheumatoid factor; SDAI, Simplified Disease Activity Index; TACERA, Towards A CurE for RA.

Values are number (percentage) or mean (standard deviation).

Of the 267 patients, 130 (48.7%), 67 (25.1%), and 70 (26.2%) were enrolled in the first, second, and third recruitment periods, respectively. No statistically significant differences were found across these three groups with respect to disease-related variables, prescribed medication, and PROMs at baseline. However, there were statistically significant differences found in the age, ethnicity, alcohol consumption, and CCI distributions, with the third group being the oldest on average (51.5 vs 53 vs 56 years old), having the highest proportion of whites (71.5% vs 62.7% vs 84.3%), lowest proportion not consuming alcohol at entry (32.3% vs 44.8% vs 20%) and lowest levels of comorbidities (CCIs of 0.52 vs 0.49 vs 0.26).

Overall, patients had moderate to severe disease at baseline as measured by both DAS28-CRP and SDAI. After baseline assessment, methotrexate (MTX) was prescribed to 75.7% of patients; 58.4% were prescribed nonmethotrexate major DMARDs (51.7% hydroxychloroquine alone, 5.7% sulfasalazine alone, and 1.1% both hydroxychloroquine and sulfasalazine); 6.4% received oral glucocorticoids (average prednisolone dose of 11.1 mg/day, range = 4–30 mg/day) and 47.2% parenteral glucocorticoids (i.e. intra-articular, intravenous, or intramuscular glucocorticoid administration). Overall, 33% were prescribed only one class/type of medication, while 66.3% were prescribed combination therapy (including with glucocorticoids) at baseline. Based on medication patterns at baseline, 26.6%, 19.9%, and 52.8% were prescribed single-RA therapy excluding glucocorticoids, dual therapy (i.e. MTX with another DMARD excluding glucocorticoids), and therapies that included glucocorticoid usage, respectively. Initial pattern of therapy did not appear to associate with either baseline X-ray scores or CCI (*p* = 0.56 and 0.23, respectively) but, as expected, was associated with disease activity measured using both SDAI and DAS28-CRP (*p* = 0.01 and 0.004, respectively). Two subjects did not receive any RA medication by the time of their first follow-up assessment and were excluded from analyses.

### Disease activity, response, and remission at 6 months

Two hundred and forty-five patients (of the 267) were followed up to or beyond their 6-month assessment visit, with 75, 2, 60, 3, and 105 having their last assessment visit at 6, 9, 12, 15, and 18 months, respectively. Of those recruited in the first, second, and third periods, 80.8% (105), 83.6% (56) and 92.9% (65) reached their target follow-up assessment visits of 18, 12, and 6 months, respectively. Of the 245 followed up to or beyond 6 months, 239 attended their 6-month assessment visit. The disease activity and disease-related outcome measures for these 239 patients are summarised in Table 2. At 6 months, the mean (SD) DAS28-CRP and SDAI were 3.04 (1.25) and 11.37 (10.71), respectively (Supplementary Table 1). Based on EULAR response criterion, 110 (47.2%) patients had good response, 79 (33.9%) moderate, and 44 (18.9%) no response. Regarding remission by different criteria, 97 (41.3%) patients achieved DAS28-CRP remission, 57 (24.3%) SDAI remission, and 51 (21.5%) met the ACR/EULAR Boolean remission criteria reflecting increased stringency of definitions. All 57 patients in SDAI remission were in DAS28-CRP remission. There was excellent agreement between SDAI and ACR/EULAR Boolean remissions (Cohen’s kappa of 0.9).

**Table 2.** Disease activity response and remission at 6 months.

Characteristics	6-month (*n* = 239)	6-month change from baseline	Missing data frequency
DAS28-CRP remission (DAS28-CRP < 2.6)^a^	97 (41.3%)		4
DAS28-ESR remission (DAS28-ESR < 2.6)^a^	92 (39.0%)		4
SDAI remission (SDAI ⩽ 3.3)^a^	57 (24.3%)		4
ACR/EULAR boolean remission^a^	51 (21.5%)		2
EULAR response^a^			6
Good response		110 (47.2%)	
Moderate response		79 (33.9%)	
No response		44 (18.9%)	

ACR, American College of Rheumatology; DAS28-CRP and DAS28-ESR, 28-joint Disease Activity Score with C-reactive protein and with erythrocyte sedimentation rate; EULAR, European League Against Rheumatism; SDAI, Simplified Disease Activity Index.

a Based on those with outcome components not missing or where the composite outcome is inferred even if some components are missing (e.g. ACR/EULAR Boolean Remission).

Other outcome measures are described in Supplementary Table 1.

### Predictors of clinical remission at 6 months

Results of the univariate logistic regression models for SDAI and DAS28-CRP used for screening baseline variables for the second-stage multivariate logistic regression analyses are shown in Supplementary Table 2. Briefly, we found that sex, alcohol consumption, disease activity, HAQ, and the SF-36 Mental Component Score screened positive for both outcomes. Ethnicity screened positive for the logistic regression model for DAS28-CRP but not for SDAI. However, in the second stage, we included all variables that screened positive in the multivariate logistic regression models for either outcomes, in addition to information on prescribed RA medication.

Multivariate logistic regression models for SDAI and DAS28-CRP remissions are shown in Table 3. In both models, 6-month clinical remission was predicted by lower functional disability and disease activity at baseline. The odds ratios related to level of disease activity at baseline are 0.65 (95% CI: 0.47–0.91) for a 10-unit change in SDAI or 0.67 (95% CI: 0.49–0.92) for a 1-unit change in DAS28-CRP, reflecting that patients with a baseline disease activity 10 (or 1) units higher have approximately a reduction of a third in the odds of achieving 6-month remission, controlling for other variables. Higher baseline functional disability was associated with a reduced likelihood of achieving SDAI remission (odds ratio (OR) = 0.90, 95% CI: 0.83–0.98 for a 0.125 increase in HAQ) or DAS28-CRP remission (OR = 0.92, 95% CI: 0.86–0.99 for a 0.125 increase in HAQ) at 6 months.

**Table 3.** Multivariate logistic regression models for SDAI and DAS28-CRP remission at 6 months.

Predictors	Log (odds ratio)	Standard error	*p* value
SDAI remission at 6 months			
Intercept	–0.169	0.597	0.777
Sex (male vs female)	–0.033	0.409	0.936
Ethnicity (others vs white)	0.407	0.439	0.353
SDAI at baseline	–0.043	0.017	0.011
HAQ at baseline	–0.818	0.328	0.013
SF-36 MCS at baseline	0.025	0.018	0.167
Alcohol consumption at baseline			0.010
1–5 units per week vs none	1.393	0.491	0.005
>5 units per week vs none	1.023	0.558	0.067
Prescribed medication at baseline^a^			0.083
MTX and other DMARDs without glucocorticoids vs therapies with glucocorticoids	0.392	0.439	0.372
Monotherapy, not glucocorticoids vs therapies with glucocorticoids	–0.706	0.435	0.105
DAS28-CRP remission at 6 months			
Intercept	1.808	0.768	0.019
Sex (male vs female)	–0.194	0.375	0.605
Ethnicity (others vs white)	–0.254	0.371	0.493
DAS28-CRP at baseline	–0.399	0.162	0.014
HAQ at baseline	–0.670	0.288	0.020
SF-36 MCS at baseline	0.027	0.016	0.085
Alcohol consumption at baseline			0.007
1–5 units per week vs none	0.936	0.382	0.014
>5 units per week vs none	1.310	0.456	0.004
Prescribed medication at baseline^a^			0.201
MTX and other DMARDs without glucocorticoids vs therapies with glucocorticoids	0.144	0.411	0.727
Monotherapy, not glucocorticoids vs therapies with glucocorticoids	–0.584	0.377	0.122

HAQ, Health Assessment Questionnaire; MTX, methotrexate; SDAI, Simplified Disease Activity Index; SF-36 MCS, Short Form 36 Health Survey Mental Component Score.

a The contrast of MTX and other DMARDs without glucocorticoids *versus* monotherapy gave Log (odds ratio) estimates of 1.099 and 0.728 with standard errors of 0.517 and 0.468 and *p* values of 0.034 and 0.120 for outcomes SDAI and DAS28-CRP, respectively.

Although both models indicated that alcohol consumption increased the odds of remission at 6 months compared to not consuming alcohol, they gave conflicting ordering of effect sizes across the alcohol consumption categories of 1–5 units and greater than 5 units per week. Moreover, in the SDAI model, there was a suggestion that being prescribed dual combination of MTX and a second DMARD increased three-fold the likelihood of SDAI remission compared to receiving monotherapy (OR = 3.00; 95% CI = 1.09–8.27; *p* = 0.034).

**Figure 1.**
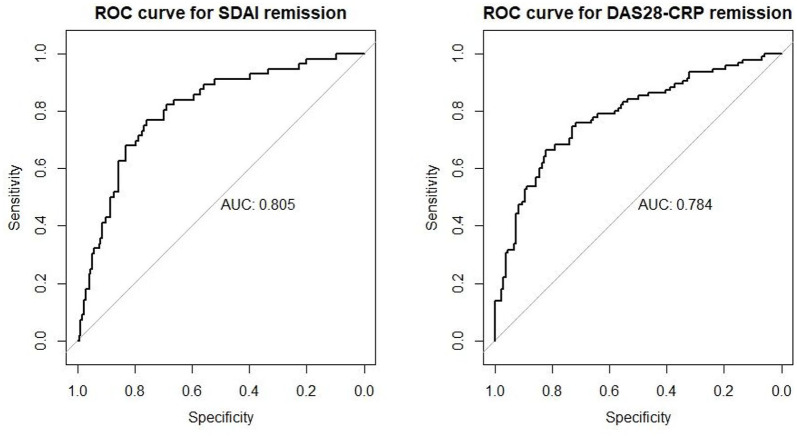
Receiver operating curves (ROCs) for SDAI and DAS28-CRP remission at 6 months.

The receiver operating characteristic (ROC) curves for these two multivariate logistic models for remission are shown in Figure 1. The areas under the ROC curves (AUCs) are 0.805 and 0.784 for SDAI and DAS2-CRP remission; indicating good overall performance.

### Characterising disease activity over time

**Figure 2.**
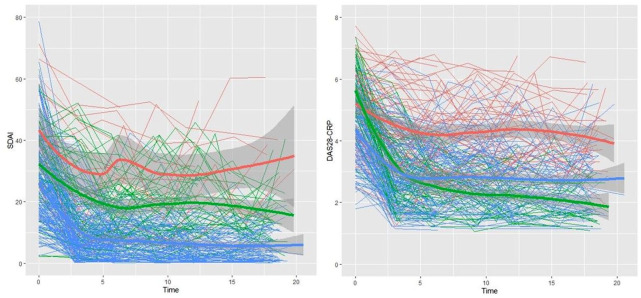
Individual and mean disease activity profiles over 18 months stratified by predicted class membership. Based on SDAI, Class1 (Red; Inadequate Response (IR)): 17 (6.5%), Class 2 (Green; higher baseline activity responder (HBAR)): 59 (22.4%), Class 3 (Blue; lower baseline activity responder (LBAR)): 187 (71.1%). Based on DAS28-CRP, Class1 (Red; IR): 57 (21.7%), Class 2 (Green; HBAR): 56 (21.3%), Class 3 (Blue; LBAR): 150 (57.0%).

Figure 2 shows the observed individual trajectories of SDAI and DAS28-CRP for the 267 patients. From the figure, there is evidence of substantive within- and between-patient variation in disease activity profiles and potential distinct trajectory subtypes.

Table 4 and Supplementary Table 3 show the results of fitting LCMMs to SDAI and DAS28-CRP, respectively, when accounting for the baseline prescribing of medication. Both models provide evidence for three distinct subpopulations or latent classes. Class 1 corresponds to an inadequate responder (IR) group that, on average, started with high baseline disease activity that early on showed improvement, in association with initial medication, and then plateaued with moderate to high levels of disease activity. Class 2 corresponds to a higher baseline activity responder (HBAR) group that, on average, started with high baseline disease activity but showed sustained improvement over time. Class 3 corresponds to a lower baseline activity responder (LBAR) group that, on average, started with moderate levels of disease activity and showed sustained improvement (see Figure 2 and Table 5). These two three-class LCMMs had lower BIC (9368.69 and 3777.78 for three-class models vs 9421.86 and 3803.88, respectively, for four-class models) and higher relative entropy (0.663 and 0.591 for three-class models vs 0.621 and 0.552) than the corresponding four-class LCMMs.

**Table 4.** Latent class mixed model for SDAI while controlling for baseline medication: 3 classes.

	Estimate	Standard error	*p* value
Multinomial class-membership model			
Class 1 (IR) vs Class 3 (LBAR)			
Intercept	–2.118	0.289	0.000
Class 2 (HBAR) vs Class 3 (LBAR)			
Intercept	–0.721	0.225	0.001
Linear mixed model			
Intercept			
Class 1	27.360	4.769	0.000
Class 2	15.972	2.593	0.000
Class 3	21.062	1.746	0.000
Disease duration, months			
Class 1	1.275	0.526	0.015
Class 2	0.675	0.223	0.002
Class 3	0.136	0.142	0.339
Sex			
Male vs Female Class 1	14.495	3.383	0.000
Male vs Female Class 2	2.039	1.800	0.257
Male vs Female Class 3	1.747	0.873	0.045
Alcohol consumption at baseline, units per week			
1–5 units per week vs none Class 1	–2.007	3.946	0.611
1–5 units per week vs none Class 2	–5.420	1.699	0.001
1–5 units per week vs none Class 3	–1.430	0.814	0.079
>5 units per week vs none Class 1	–11.588	5.035	0.021
>5 units per week vs none Class 2	–4.772	1.738	0.006
>5 units per week vs none Class 3	–2.641	1.070	0.014
HAQ score at baseline			
Class 1	5.639	1.417	0.000
Class 2	9.897	0.948	0.000
Class 3	4.070	0.557	0.000
Centred SF-36 Mental Component Score at baseline			
Class 1	–0.309	0.114	0.007
Class 2	0.090	0.060	0.133
Class 3	–0.059	0.035	0.095
Follow-up time (in months) within 5 months			
Class 1	–2.456	0.819	0.003
Class 2	–2.346	0.444	0.000
Class 3	–4.005	0.260	0.000
Follow-up time (in month) after 5 months			
Class 1	0.837	0.265	0.002
Class 2	0.112	0.145	0.440
Class 3	0.047	0.089	0.595
Prescribed other DMARDs and MTX, not glucocorticoids vs therapies with glucocorticoids at baseline			
Class 1	–18.742	2.775	0.000
Class 2	–7.456	1.775	0.000
Class 3	–0.838	1.331	0.529
Prescribed monotherapy, not glucocorticoids v therapies with glucocorticoids at baseline			
Class 1	5.398	3.394	0.112
Class 2	–1.905	1.475	0.197
Class 3	0.662	0.890	0.457
Variance components			
Variance of random intercept	74.947		
Variance of random slope within 5 months	3.256		
Variance of random slope after 5 months	0.050		
Error standard deviation	7.600	0.215	

HAQ, Health Assessment Questionnaire; HBAR, higher baseline activity responder; IR, inadequate responder; LBAR, lower baseline activity responder; MTX, methotrexate; SDAI, Simplified Disease Activity Index; SF-36, Short Form 36 Health Survey.

**Table 5.** Disease outcomes by allocated SDAI (first row of cell) and DAS28-CRP (second row of cell) classes, controlling for baseline medication.

	Class 1 (IR)	Class 2 (HBAR)	Class3 (LBAR)	*p* value
Baseline SDAI (SD)	44.65 (17.50)	32.08 (11.99)	26.44 (13.66)	<0.0001
	33.63 (16.28)	38.83 (12.95)	23.23 (11.05)	<0.0001
SDAI at 6 months (SD)^a^	32.84 (14.10)	18.74 (8.97)	7.36 (6.82)	<0.0001
	21.71 (13.14)	6.85 (6.38)	9.14 (8.29)	<0.0001
SDAI 6-month change from baseline	–9.11 (18.70)	–12.83 (12.74)	–19.37 (14.12)	0.002
	–11.00 (13.92)	–33.28 (13.94)	–13.76 (10.04)	<0.0001
DAS28-CRP (baseline)	5.89 (1.29)	5.19 (1.03)	4.65 (1.22)	<0.0001
	5.22 (1.24)	5.73 (0.89)	4.38 (1.11)	<0.0001
DAS28-CRP (6 months)	5.12 (1.23)	3.99 (1.02)	2.58 (0.93)	<0.0001
	4.22 (1.27)	2.50 (0.81)	2.79 (1.08)	<0.0001
DAS28-CRP 6-month change from baseline	–0.55 (1.37)	–1.19 (1.15)	–2.11 (1.29)	<0.0001
	–0.92 (1.15)	–3.32 (1.08)	–1.59 (1.04)	<0.0001
X-ray Score (baseline)	5.65 (5.88)	6.12 (10.83)	6.80 (8.05)	0.786
	6.11 (9.52)	7.23 (8.51)	6.50 (8.31)	0.779
X-ray annualised progression rate	1.00 (2.30)	1.08 (2.86)	0.89 (2.61)	0.898
	1.39 (3.64)	0.79 (2.04)	0.80 (2.32)	0.372
EQ5D Score (baseline)	0.33 (0.38)	0.43 (0.31)	0.52 (0.32)	0.030
	0.43 (0.33)	0.45 (0.34)	0.52 (0.32)	0.094
EQ5D Score (6 months)^a^	0.50 (0.34)	0.59 (0.27)	0.77 (0.21)	<0.0001
	0.63 (0.27)	0.79 (0.18)	0.72 (0.25)	0.003
EQ5D 6-month change from baseline	0.11 (0.25)	0.15 (0.33)	0.25 (0.31)	0.049
	0.18 (0.27)	0.36 (0.35)	0.18 (0.29)	0.002
HAQ (baseline)	1.41 (0.80)	1.44 (0.72)	1.13 (0.76)	0.015
	1.21 (0.72)	1.29 (0.81)	1.20 (0.76)	0.748
HAQ (6 months)^a^	1.10 (0.94)	1.15 (0.80)	0.50 (0.58)	<0.0001
	0.93 (0.83)	0.50 (0.61)	0.65 (0.68)	0.008
HAQ 6-month change from baseline	–0.19 (0.77)	–0.25 (0.63)	–0.63 (0.60)	0.0001
	–0.23 (0.60)	–0.82 (0.81)	–0.52 (0.52)	<0.0001
SF-36 MCS (baseline)	40.2 (11.9)	43.1 (11.7)	46 (11.3)	0.05
	44.4 (11.4)	46.5 (12.2)	44.6 (11.3)	0.632
SF-36 MCS (6 months)^a^	41.2 (10.3)	46.2 (13.3)	50.9 (10.5)	0.0006
	46.4 (11.7)	55.1 (7.4)	48.3 (12.0)	0.0002
SF-36 MCS 6-month change from baseline	0.15 (11.67)	3.05 (12.36)	4.65 (9.74)	0.217
	1.54 (10.68)	7.61 (10.25)	3.64 (10.23)	0.012

EQ5D, EuroQoL five dimensions questionnaire; HAQ, Health Assessment Questionnaire; HBAR, higher baseline activity responder; IR, inadequate responder; LBAR, lower baseline activity responder; MCS, Short Form 36 Health Survey Mental Component Score; SD, standard deviation; SDAI, Simplified Disease Activity Index; SF-36, Short Form 36 Health Survey.

Mean (SD) are reported with *p* values based on ANOVA test.

a Number of patients at 6 months differs from the number at baseline within a class.

The IR, HBAR, and LBAR groups were estimated to comprise 6.5%, 22.4%, and 71.1% of the patients based on the SDAI model and 21.7%, 21.3%, and 57% of the patients based on the DAS28-CRP model. No overall statistically significant differences in mean age at entry, disease duration, SF-36 Physical Component Score at entry, sex, smoking status, alcohol consumption, and serology distributions were found across latent classes. However mean body mass index (BMI; *p* = 0.064), mean baseline levels of functional disability (*p* = 0.015), and SF-36 Mental Component Scores (*p* = 0.05) either were close to being or were statistically significantly different across the three classes/groups based on the SDAI model but not DAS28-CRP model. Table 5 shows that based on the SDAI model, the LBAR group had on average significantly less functional disability than the other groups. A similar pattern was seen for mental health, with, on average, better mental health scores seen in the LBAR group (mean of 46) compared to both IR and HBAR (means of 40.2 and 43.1, respectively). Mean BMI was approximately 3.5 kg/m^2^ higher in the IR group compared to the other SDAI groups. No evidence was found that prescribing behaviour of clinicians varied across the SDAI or DAS28-CRP defined groups (see Supplementary Table 4).

In the IR group, lower disease activity levels over time (measured by SDAI) were associated with being female, shorter symptom duration, consuming greater than 5 units of alcohol per week, less functional disability, and better mental well-being at baseline. Being prescribed dual therapy of MTX and a second DMARD at baseline was associated with lower SDAI over time compared to either receiving monotherapy (excluding glucocorticoids) or therapies that included glucocorticoid usage (see Table 4).

For the HBAR group, lower levels of SDAI over time were associated with shorter symptom duration, increasing levels of alcohol consumption, and less functional disability. Moreover lower levels of disease activity were associated with being prescribed dual therapy of MTX and a second DMARD at baseline. In the LBAR group, being female, consuming alcohol and lower level of functional disability at baseline were associated with lower levels of SDAI.

Based on the DAS28-CRP model (Supplementary Table 2), lower levels of functional disability, better mental well-being and dual therapy of MTX with a second DMARD at baseline were associated with lower DAS28-CRP over time in the IR group. Higher levels of functional disability and receiving monotherapy (excluding glucocorticoids) were associated with higher levels of disease activity in the HBAR group. While being female, drinking alcohol, having lower levels of functional disability and better mental health were associated with lower DAS28-CRP over time in the LBAR group.

Table 5 summarises other patient outcome data (X-ray score, EQ-5D, HAQ, and SF-36 MCS) by allocated latent trajectory class. Class assignment could be made for 263 of the 267 eligible patients with some baseline and outcome information. The average EQ-5D scores both at baseline and 6 months were significantly higher in the LBAR group than the other groups as defined by the SDAI model (*p* < 0.0001 and 0.049), while average level of functional disability remained significantly lower in this group at 6 months (*p* < 0.0001). There was no statistical evidence for a difference in average X-ray annualised progression rate across groups.

## Discussion

The TACERA cohort provides a unique opportunity to follow newly diagnosed and initially medication naïve patients in the United Kingdom, whose clinical outcomes are linked to extensive and detailed biological phenotyping (to be reported separately). We focused solely on seropositive RA patients whom have few nonsevere comorbidities to reduce heterogeneity and to identify factors associated with outcomes in this subset.

We found clear evidence that remission at 6 months, whether defined using SDAI or DAS28-CRP, was associated with lower levels of disease activity and functional disability at baseline and with alcohol consumption at baseline. The findings that disease activity and functional disability are negatively associated with achieving remission have been reported before.^28,30^ Aletaha and colleagues^35^ have previously shown that disease activity early in the course of treatment predicts response to therapy after 1 year. However, other variables previously identified as associated with remission, such as age, sex, ethnicity, and RA medications were not found to be statistically significant in our study. This may be due to the power of this study (especially when restricted to the outcome defined at a single time point), to the use of a treat-to-target medication strategy, or to the fact that some variables may be less important in a pure seropositive disease cohort.

We also identified three trajectories of disease course and potential factors associated with them and also with disease activity levels within trajectories. We used the same trajectory labels (IR, HBAR, and LBAR) in both SDAI and DAS28-CRP models, but the estimated proportion of patients who fell into the three groups varied according to the way disease activity was measured. Moreover, the agreement between these two classifications was weak (Cohen’s Kappa of 0.18); and significant overall associations of baseline functional disability and SF-36 mental component score and near significant association with BMI were found only with the SDAI trajectories; thus suggesting these disease activity indices are not interchangeable.

Interestingly, we found no evidence for differences in prescribing behaviour at baseline among the trajectory classes irrespective of how trajectory classifications were made. However, we found clear evidence that, even as early as 6 months, disease outcomes (e.g. EQ-5D, HAQ) and their changes from baseline differed between trajectory groups, with the IR group generally having the least improvement and poorer health outcomes. No evidence for differing annualised X-ray progression rates were seen across groups. This may be expected, given the short length of follow-up and early-intensive management. The findings of very few if any significant associations between standard clinical measures and trajectory classes, evidence of clear changes in outcomes as early as 6 months and relative entropy values (0.663 and 0.591) in our latent class models that indicate further improvements can be made would possibly suggest that these trajectories may additionally be linked to distinct immunopathogenic subsets. Thus immunological biomarkers at baseline and early in follow-up (before 6 months) may be useful in identifying patients who beyond 6 months will have inadequate response to initial synthetic disease-modifying treatment. This hypothesis is speculative at present. However, the RA-MAP Project was established towards this goal, and we are currently investigating these trajectories from a biological viewpoint, using the extensive biological data set collected in TACERA.

Heterogeneity in the effects of covariates on average SDAI level over time was seen across trajectories. Specifically, we found that effect sizes differed across trajectories with respect to functional disability, mental health, alcohol consumption, and combination MTX with second DMARD compared to therapies with glucocorticoid usage. This provides evidence to support a stratified approach to management and treatment of patients falling into different trajectory groups. For example, our models could be used to assign probabilities of different disease activity responses at 6 months or other time points to different treatment options, such as disease-modifying monotherapy, dual therapies without steroids, or therapies that include steroids, to decide clinically over a particular clinically relevant time horizon what would be the optimal treatment to assign a patient who presents with moderate or high levels of disease activity. In addition, the predictions of trajectory classes or the likely responses to change in treatments could be dynamically updated and refined at follow-up visits using explanatory variables and outcome information.

Our results concerning the effect of alcohol consumption on both SDAI remission at 6 months and disease activity levels within SDAI trajectories suggest that consuming alcohol is associated with higher remission rates and lower disease activity over time. Similar inverse associations have been reported by other studies.^36–40^ These findings may be due to alcohol intake being associated with lower levels of inflammatory markers and possibly because certain alcoholic drinks, for example, red wine, may be antioxidant due to their flavonoid content, counteracting postprandial oxidative stress.^41,42^

The findings that mental health is a predictor of disease activity over time in the IR group and also possibly the LBAR group but not the HBAR group are worth highlighting. One-year results from the Scottish Early Rheumatoid Arthritis Inception (SERA) cohort have identified that predictors of functional disability at 1 year appear to be dominated by psychosocial rather than more traditional clinical measures, emphasising the potential benefit which may result from early access to nonpharmacological interventions targeting key psychosocial factors, such as mental health and work disability.^43^ The possibility of adopting a nonpharmacological intervention strategy for those in the IR and LBAR (that make up approximately 78% of the RA patients) should be explored in more depth.

Latent class mixed modelling approaches have only recently begun to be used to investigate whether distinct subpopulations exist within RA with respect to disease course.^5,44,45^ Previous studies using DAS28 have identified similar trajectory groups thus suggesting that our latent classes have external validity. The larger study by Barnabe and colleagues was able to identify five trajectory groups instead of three. However, combining their Groups 1 and 4 (i.e. both with initial high activity) into a single group would coincide with our HBAR group, while combining their Groups 2 and 3 (i.e. both with initial moderate activity) would correspond to our LBAR group. In work that we have done in RA-MAP using individual participant data from multiple clinical trials, we were also able to identify three distinct latent DAS28-ESR trajectories in both baseline methotrexate-naïve treated patients and methotrexate-exposed patients.^29^ The baseline methotrexate-naïve patients in the trials reflected a relatively early in disease population which is more comparable to our TACERA cohort. It thus was reassuring that similar latent trajectory classes were identified from TACERA using both DAS28-CRP and SDAI. Our work therefore demonstrates that similar trajectory groups exist within a purely seropositive population with low levels of comorbidities. In addition, we controlled for factors that may impact on disease activity over time within these trajectory groups.

The decision to restrict to an inception cohort with low levels of comorbidities was made to reduce variation due to comorbidities when addressing the RA-MAP Project’s primary objective of understanding immune function and response in RA through use of an extensive set of biological markers, measured at baseline and over time. This decision has implications for generalisability to the newly diagnosed RA population in the United Kingdom, especially those with significant comorbidities.

## Conclusion

In conclusion, we found that in early seropositive RA with few nonsevere comorbidities, lower baseline levels of functional disability, and disease activity, along with alcohol intake, are associated with 6-month clinical remission. We identified three subpopulations based on disease trajectories that not only differ in terms of disease course but also with regard to the effect of risk factors, such as mental well-being, on disease activity over time. Our data further highlight RA heterogeneity (i.e. trajectory classes) not explainable by clinical factors and indicate the possible use of biomarkers collected at baseline and early follow-up to help patient management and better targeting of existing and novel therapies.

## Supplemental Material

sj-docx-1-tab-10.1177_1759720X211043977 – Supplemental material for Characterization of disease course and remission in early seropositive rheumatoid arthritis: results from the TACERA longitudinal cohort study Click here for additional data file. Supplemental material, sj-docx-1-tab-10.1177_1759720X211043977 for Characterization of disease course and remission in early seropositive rheumatoid arthritis: results from the TACERA longitudinal cohort study by RA-MAP Consortium, Michael Barnes, Sarah Brockbank, Ian N Bruce, Coziana Ciurtin, Andrew P. Cop, Michael R. Ehrenstein, Paul Emery, Benjamin A. Fisher, John Isaacs, Ruth Matthews, Iain B. McInnes, Hayley Noble, Ayako Wakatsuki Pedersen, Costantino Pitzalis, Karim Raza, Anthony Rowe, Gemma Simpson, Dominic Stringer, Peter C. Taylor, Brian Tom and Yujie Zhong in Therapeutic Advances in Musculoskeletal Disease

sj-docx-2-tab-10.1177_1759720X211043977 – Supplemental material for Characterization of disease course and remission in early seropositive rheumatoid arthritis: results from the TACERA longitudinal cohort study Click here for additional data file. Supplemental material, sj-docx-2-tab-10.1177_1759720X211043977 for Characterization of disease course and remission in early seropositive rheumatoid arthritis: results from the TACERA longitudinal cohort study by Michael Barnes, Sarah Brockbank, Ian N Bruce, Coziana Ciurtin, Andrew P. Cop, Michael R. Ehrenstein, Paul Emery, Benjamin A. Fisher, John Isaacs, Ruth Matthews, Iain B. McInnes, Hayley Noble, Ayako Wakatsuki Pedersen, Costantino Pitzalis, Karim Raza, Anthony Rowe, Gemma Simpson, Dominic Stringer, Peter C. Taylor, Brian Tom and Yujie Zhong in Therapeutic Advances in Musculoskeletal Disease
