# Supplementary material for: Characterization of disease course and remission in early seropositive rheumatoid arthritis: results from the TACERA longitudinal cohort study
Source: Ther Adv Musculoskelet Dis. 2021 Oct 21;13:1759720X211043977. doi: 10.1177/1759720X211043977 (PMC8544781; doi:10.1177/1759720X211043977)
Supplement: sj-docx-1-tab-10.1177_1759720X211043977 – Supplemental material for Characterization of disease course and remission in early seropositive rheumatoid arthritis: results from the TACERA longitudinal cohort study [file sj-docx-1-tab-10.1177_1759720X211043977.docx]

| **Paper title** |
| --- |
| **Characterization of disease course and remission in early seropositive rheumatoid arthritis: Results from the TACERA longitudinal cohort study** |
| **Author** |
| **RA-MAP Consortium** |
| **Corresponding Authors** |
| **Brian Tom and Andrew Cope** |

| **List of Contributing Authors (for REF purposes only)** – in alphabetical order | | | |
| --- | --- | --- | --- |
| **Name** | **Affiliation** | **Please provide details of contribution** | **Competing interest statement (if any)** |
| **Sarah Brockbank** | Institute of Cellular Medicine, Newcastle University | Contribution to experimental design, acquisition, analysis and the data analysis plan. Operational contribution to drafting and approval of the manuscript. Agree to be accountable for all aspects of the work | None |
| **Michael Barnes** | Centre for Translational Bioinformatics  William Harvey Research Institute  Barts and The London School of Medicine and Dentistry  Queen Mary University of London  John Vane Science Centre  Charterhouse Square  London  EC1M 6BQ | Chaired data analysis team, contributed to experimental design, reviewed manuscript | None |
| **Ian N Bruce** | Arthritis Research UK Centre for Epidemiology, Faculty of Biology, Medicine and Health, The University of Manchester and NIHR Manchester Biomedical Research Centre, Manchester University Hospitals NHS Foundation Trust, Manchester Academic Health Science Centre, Manchester, UK | Contributed to the study design, interpretation of data analysis and drafting and final manuscript approvals. Agrees to be accountable for this work | None |
| **Andrew P. Cope** | Academic Department of Rheumatology, Division of Immunology, Infection and Inflammatory Disease, Faculty of Life Sciences, King’s College London | Workpackage Lead for this study: contributions to study set up, acquisition and provision of datasets and analysis, and critical review of the manuscript. Agree to be accountable for all aspects of the work | None |
| **Coziana Ciurtin** | Department of Rheumatology, University College London Hospital, 3rd Floor Central, 250 Euston Road, London, NW1 2PG | Contribution to data acquisition (1), revising critically the work (2), final approval of the version to be published (3) and agreement to be accountable for all aspects of the work. | None |
| **Michael R Ehrenstein** | Division of Medicine, UCL | Substantial contribution to the conception and design, analysis and interpretation. I have read the manuscript and approve its content. It has taken into consideration all the findings that I have had discussed at length with the RA-MAP group at the PMB meertings | None |
| **Paul Emery** | Arthritis Research UK,  Professor of Rheumatology  Director Leeds BRC, The LTHT  Leeds Institute Rheumatic and Musculoskeletal Medicine University Leeds | Contribution to study design, patient recruitment and reviewing and approving manuscript | None |
| **Benjamin A Fisher** | 1.National Institute for Health Research (NIHR) Birmingham Biomedical Research Centre  2.Rheumatology Research Group, Institute of Inflammation and Ageing, University of Birmingham, Birmingham, UK  3.Rheumatology Department, University Hospitals Birmingham NHS Foundation Trust, Birmingham, UK | Contribution to study design, acquisition and interpretation of data, revision of manuscript, and approval of final manuscript. | Contribution to study design, acquisition and interpretation of data, revision of manuscript, and approval of final manuscript. |
| **John Isaacs** | Institute of Cellular Medicine, Newcastle University and NIHR Newcastle Biomedical Research Centre at Newcastle upon Tyne Hospitals NHS Foundation Trust and Newcastle University, Newcastle upon Tyne, UK. | Chief investigator RA-MAP consortium. Major contributions to concept and design, drafting and revising the manuscript, final approvals, and accountability for all aspects of the work. | None |
| **Iain B. McInnes** | Institute of Infection, Immunity and Inflammation, College of Medical, Veterinary and Life Sciences, University of Glasgow, Glasgow, UK | Contribution to the study concept; critical review and approval of manuscript. Agree to be accountable for all aspects of the work | Received research funding or honoraria from AbbVie, BMS, UCB, Pfizer, Novartis and Jansen |
| **Ruth Matthews** | Clinical Trial Manager  Drug Development Unit  The Institute of Cancer Research  15 Cotswold Road  Sutton  SM2 5NG | Substantial contributions to the study design and implementation, acquisition and analysis (data quality) of the work  Final approval for the version to be published  Agree to be accountable for all aspects of the work | None |
| **Hayley Noble** | Clinical Trial Manager  Neurosciences Research Centre  Molecular & Clinical Sciences Research Institute  St Georges, University of London  Cranmer Terrace  London  SW17 0RE | Substantial contributions to the acquisition and analysis (data quality) of the work  Final approval for the version to be published  Agree to be accountable for all aspects of the work | None |
| **Costantino Pitzalis** | Queen Mary University of London | Conceiving the study, supervising the clinical trial team and recruiting patients | None |
| **Ayako Wakatsuki Pedersen** | Institute of Cellular Medicine, Newcastle University | Contribution to experimental design, acquisition, analysis and the data analysis plan. Operational contribution to drafting and approval of the manuscript. Agree to be accountable for all aspects of the work | None |
| **Karim Raza** | Arthritis Research UK Centre for Rheumatoid Arthritis Pathogenies ad MRC / Arthritis Research UK Centre for Musculoskeletal Ageing, Institute of Inflammation and Ageing, University of Birmingham, Birmingham AND department of Rheumatology Sandwell and West Birmingham Hospitals NHS Trust, Birmingham | Contribution to study design, patient recruitment and reviewing and approving manuscript | Karim Raza has received honoraria from Pfizer, Lilly, UCB, BMS, Janssen and Roche, and grants from AbbVie, outside the submitted work |
| **Anthony Rowe** | Janssen Research & Development, LLC, Spring House, Pennsylvania, USA | - Conception & Design of the RA-MAP consortium data analysis working group including design of data analysis strategy  - Design of consortium data management and analysis platform  -Drafting and Review of Manuscript | Employee and shareholder in the Johnson and Johnson family of companies |
| **Gemma Simpson** | TACERA Study Coordinator  Kings College London | Substantial contributions to the study design and implementation, acquisition and analysis (data quality) of the work  Final approval for the version to be published  Agree to be accountable for all aspects of the work | None |
| **Dominic Stringer** | Department of Biostatistics and Health Informatics, Institute of Psychiatry, Psychology and Neuroscience, King’s College London, London, UK | Substantial contributions to design of data capture tools, acquisition and collation of the data. Reviewed and approve final manuscript. | None |
| **Peter C. Taylor** | Norman Collisson Professor of Musculoskeletal Sciences  Head of Clinical Sciences  Botnar Research Centre Nuffield Department of Orthopaedics, Rheumatology and Musculoskeletal Sciences, University of Oxford  Botnar Research Centre, Windmill Road, Headington,  Oxford,  OX3  7LD. | Contribution to the study concept, acquisiti0on and interpretation of data; critical revision of manuscript | None |
| **Brian Tom** | MRC Biostatistics Unit, University of Cambridge | Design of the work, data acquisition and cleaning, statistical analysis, interpretation and writing; approval of final version of manuscript and accountable for all aspects of the work | None |
| **Yujie Zhong** | MRC Biostatistics Unit, University of Cambridge | Statistical analysis, interpretation and revising manuscript critically; approval of final version of manuscript and accountable for all aspects of the work | None |
