# Supplementary material for: Characterization of disease course and remission in early seropositive rheumatoid arthritis: results from the TACERA longitudinal cohort study
Source: Ther Adv Musculoskelet Dis. 2021 Oct 21;13:1759720X211043977. doi: 10.1177/1759720X211043977 (PMC8544781; doi:10.1177/1759720X211043977)
Supplement: sj-docx-2-tab-10.1177_1759720X211043977 – Supplemental material for Characterization of disease course and remission in early seropositive rheumatoid arthritis: results from the TACERA longitudinal cohort study [file sj-docx-2-tab-10.1177_1759720X211043977.docx]

**Running Title**:

Characterization of disease course and remission in early seropositive rheumatoid arthritis

**Title:**

Characterization of disease course and remission in early seropositive rheumatoid arthritis: Results from the TACERA longitudinal cohort study

**Authors:**

The RA-MAP Consortium,

United Kingdom

**Correspondence to:**

Dr Brian Tom,

MRC Biostatistics Unit,

University of Cambridge,

United Kingdom

e-mail: [brian.tom@mrc-bsu.cam.ac.uk](mailto:brian.tom@mrc-bsu.cam.ac.uk)

**SUPPLEMENTARY MATERIAL**

**SAMPLE SIZE**

The original sample size calculation for TACERA of 410 enrolled patients with early RA was based on estimating the 6-month clinical remission rate. It used the recommendation by Peduzzi et al. (1) of 10 patient outcomes per variable in regression analyses, an estimated 6-month clinical remission rate of 40% (based on DAS28-CRP remission criteria), a margin of error of ± 5% and anticipated a 10% loss to follow-up. This sample size was revised to 274 patients after re-assessment informed by data on the first 21 patients and applying the methodology of Dobbin and Simon (2) to develop a classifier for prediction of remission rather than estimating the 6-month remission rate.

**REFERENCES**

1. Peduzzi P, Concato J, Kemper E, Holford TR, Feinstein AR. A simulation study of the number of events per variable in logistic regression analysis. Journal of clinical epidemiology 1996;49(12):1373-9.
2. Dobbin KK, Simon RM. Sample size planning for developing classifiers using high-dimensional DNA microarray data. Biostatistics 2007;8(1):101-17.

**TABLES**

Supplementary Table 1: Baseline and 6-month characteristics of disease measures in TACERA Study

| **Characteristics** | **Baseline (n=267)** | **6-month (n=239)** | **6-month Change from baseline** | **p-value** |
| --- | --- | --- | --- | --- |
| X-ray Annualised Progression (v1) ** |  |  | 0.96 (2.63) |  |
| X-ray Annualised Progression (v2)** |  |  | 1.03 (2.67) |  |
| TJC28 | 9.82 (6.80) | 3.97 (5.26) | -5.75 (6.68) | < 0.001 |
| SJC28 | 6.98 (5.19) | 1.97 (2.87) | -5.08 (5.26) | < 0.001 |
| TJC66 | 16.28 (12.07) | 6.56 (9.79) | -9.60 (11.63) | < 0.001 |
| SJC66 | 9.19 (7.43) | 2.45 (3.44) | -6.95 (7.25) | < 0.001 |
| ESR (mm/h) | 31.36 (25.06) | 16.46 (15.27) | -14.03 (22.38) | < 0.001 |
| CRP (mg/l) | 17.39 (22.89) | 6.47 (9) | -10.57 (20.31) | < 0.001 |
| Patient Global Assessment | 53.10 (26.92) | 25.78 (24.9) | -27.33 (30.49) | < 0.001 |
| Evaluator Global Assessment | 50.21 (22.93) | 20.82 (21) | -28.96 (26.81) | < 0.001 |
| DAS28-ESR | 5.20 (1.38) | 3.13 (1.45) | -2.04 (1.54) | < 0.001 |
| DAS28-CRP | 4.85 (1.22) | 3.04 (1.25) | -1.82 (1.36) | < 0.001 |
| SDAI | 28.80 (14.29) | 11.37 (10.71) | -17.32 (14.48) | < 0.001 |
| HAQ | 1.22 (0.76) | 0.67 (0.71) | -0.52 (0.64) | < 0.001 |
| EQ-5D | 0.49 (0.32) | 0.72 (0.25) | 0.22 (0.31) | < 0.001 |
| SF-36 PCS (Physical) | 37.21 (9.03) | 43.95 (9.69) | 6.60 (8.56) | < 0.001 |
| SF-36 MCS (Mental) | 44.94 (11.51) | 49.38 (11.42) | 4.06 (10.48) | < 0.001 |

The p-value corresponds to test of null hypothesis of no change from baseline
*Based on those with outcome not missing

Supplementary Table 2: Results from the Univariate Logistic Regression Models for Disease Remission (first row SDAI; second row DAS28-CRP) from Stage 1.

| Baseline Predictors | Log (Odds Ratio) | StandardError | p-value |
| --- | --- | --- | --- |
| **Remission at 6 months** |  |  |  |
| Age, years | -0.0021 0.0025 | 0.0105 0.0092 | 0.842 0.785 |
| Sex (M v F) | 0.5787  0.6004 | 0.3370  0.3091 | **0.086**  **0.052** |
| Ethnicity (Others v White) | -0.1301  -0.5525 | 0.3455  0.3089 | 0.707  **0.074** |
| Smoking  Previous v Never  Current v Never | 0.3577  0.0943  -0.2877  -0.4122 | 0.3468  0.3072  0.4334  0.3616 | 0.268 0.346  0.302  0.759  0.507  0.254 |
| Disease Duration, years | 0.6394  0.4519 | 0.6774  0.6040 | 0.345  0.454 |
| BMI, kg/m^2^ | -0.0185  -0.0168 | 0.0258  0.0222 | 0.472  0.449 |
| Obesity (Obese v Not Obese) | -0.0969  -0.1116 | 0.3394  0.2955 | 0.775  0.706 |
| Serology  RF positive only vs Both positive  ACPA positive vs Both positive | -0.0178  -0.3821  0.1660  0.3111 | 0.4425  0.3997  0.5548  0.4956 | 0.956  0.472  0.968  0.339  0.765  0.530 |
| Alcohol consumption  1-5 units per week v None  >5 units per week v None | 1.1199  0.8773  1.3260  1.6635 | 0.4206  0.3406  0.4538  0.3864 | **0.004**  **<0.001**  **0.008**  **0.010**  **0.003**  **<0.001** |
| HAQ at Baseline | -1.2251  -1.1763 | 0.2528  0.2174 | **<0.001**  **<0.001** |
| Disease Activity | -0.0614  -0.6589 | 0.0142  0.1301 | **<0.001**  **<0.001** |
| SF-36 Mental Component Score (MCS) | 0.0478  0.0463 | 0.0155  0.0133 | **0.002**  **<0.001** |
| X-ray Larsen’s Score | -0.0046  0.0173 | 0.0189  0.0161 | 0.806  0.283 |
| Prescribed medication at baseline*  MTX and other DMARDs without glucocorticoids v therapies with glucocorticoids  monotherapy, not glucocorticoids v therapies with glucocorticoids | 0.6057  0.4637  -0.1859  -0.0562 | 0.3787  0.3514  0.3881  0.3241 | 0.171  0.350  0.110  0.187  0.632  0.862 |
|  |  |  |  |

Supplementary Table 3: Latent Class Mixed Model for DAS28-CRP: 3 Classes

| **Multinomial Class Membership Model** | **Estimate** | **Standard Error** | **p-value** |
| --- | --- | --- | --- |
| Class 1 (IR) v Class 3 (LBAR) |  |  |  |
| Intercept | -0.850 | 0.244 | 0.000 |
| Class 2 (HBAR) v Class 3 (LBAR) |  |  |  |
| Intercept | -0.781 | 0.329 | 0.018 |
| **Linear Mixed Model** | **Estimate** | **Standard Error** | **p-value** |
| Intercept  Class 1  Class 2  Class 3 | 3.858  4.941  3.232 | 0.277  0.377  0.228 | 0.000  0.000  0.000 |
| Disease duration, months  Class 1  Class 2  Class 3 | 0.017  -0.003  0.026 | 0.032  0.038  0.024 | 0.599  0.932  0.288 |
| Sex  Male v Female Class 1  Male v Female Class 2  Male v Female Class 3 | 0.255  -0.034  0.313 | 0.211  0.200  0.125 | 0.228  0.867  0.013 |
| Alcohol consumption, units per week  1-5 units per week vs None Class 1  1-5 units per week vs None Class 2  1-5 units per week vs None Class 3  >5 units per week vs None Class 1  >5 units per week vs None Class 2  >5 units per week vs None Class 3 | 0.285  -0.221  -0.171  -0.266  -0.185  -0.372 | 0.192  0.193  0.141  0.227  0.280  0.177 | 0.139  0.252  0.226  0.242  0.510  0.036 |
| HAQ score at baseline  Class 1  Class 2  Class 3 | 0.939  0.402  0.863 | 0.137  0.137  0.085 | 0.000  0.003  0.000 |
| Centred SF-36 Mental component score at baseline  Class 1  Class 2  Class 3 | -0.017  0.013  -0.021 | 0.008  0.009  0.005 | 0.033  0.159  0.000 |
| Follow-up time (in month) within 5 months  Class 1  Class 2  Class 3 | -0.168  -0.624  -0.330 | 0.041  0.050  0.030 | 0.000  0.000  0.000 |
| Follow-up time (in month) after 5 months  Class 1  Class 2  Class 3 | 0.017  -0.008  0.011 | 0.019  0.027  0.014 | 0.377  0.772  0.429 |
| Prescribed other DMARDs and MTX, not glucocorticoids v therapies with glucocorticoids at baseline  Class 1  Class 2  Class 3 | -0.711  -0.323  -0.156 | 0.250  0.226  0.199 | 0.004  0.152  0.434 |
| Prescribed monotherapy, not glucocorticoids v therapies with glucocorticoids at baseline  Class 1  Class 2  Class 3 | 0.053  0.770  0.016 | 0.263  0.339  0.146 | 0.840  0.023  0.913 |
| Variance Components  Variance of Random Intercept  Variance of Random Slope within 5 months  Variance of Random Slope after 5 months  Error Standard Deviation | 0.077  0.0002  0.002  0.827 | 0.022 |  |

Supplementary Table 4: Description of select baseline characteristics by each predicted latent group based on SDAI (1^st^ row of cell) and DAS28-CRP (*2^nd^ row of cell*) while controlling for baseline medication

|  | **Class 1 (IR)** | **Class 2 (HBAR)** | **Class3 (LBAR)** | **p-value** |
| --- | --- | --- | --- | --- |
| Mean BMI (SD), kg/m^2^ | 30.78 (6.99)  *28.37 (5.97)* | 27.23 (7.03)  *27.22 (5.23)* | 27.21 (5.59)  *27.17 (6.40)* | 0.064  *0.426* |
| Alcohol consumption  None  1-5 units per week  6 and more units per week | 4 (23.5%)  *23 (40.4%)*  11 (64.7%)  *19 (33.3%)*  2 (11.8%)  *15 (26.3%)* | 24 (40.7%)  *18 (32.1%)*  21 (35.6%)  *25 (44.6%)*  14 (23.7%)  *13 (23.2%)* | 57 (30.5%)  *44 (29.3%)*  81 (43.3%)  *69 (46%)*  49 (26.2%)  *37 (24.7%)* | 0.247  *0.511* |
| Smoking status  Never  Previous  Current | 7 (41.2%)  *20 (35.1%)*  6 (35.3%)  *20 (35.1%)*  4 (23.5%)  *17 (29.8%)* | 22 (37.3%)  *20 (35.7%)*  15 (25.4%)  *25 (44.6%)*  22 (37.3%)  *11 (19.6%)* | 65 (34.8%)  *54 (36.0%)*  81 (43.3%)  *57 (38.0%)*  41 (21.9%)  *39 (26.0%)* | 0.081  *0.753* |
| MTX prescribed | 14 (82.4%)  *47 (82.5%)* | 45 (76.3%)  *43 (76.8%)* | 141 (75.4%)  *109 (72.7%)* | 0.908  *0.337* |
| Other DMARDs prescribed | 9 (52.9%)  *31 (54.4%)* | 34 (57.6%)  *36 (64.3%)* | 113 (60.4%)  *89 (58.3%)* | 0.765  *0.559* |
| Parenteral glucocorticoids prescribed | 8 (47.1%)  *30 (52.6%)* | 30 (50.8%)  *28 (50.0%)* | 87 (46.5%)  *67 (44.7%)* | 0.850  *0.544* |
| Oral glucocorticoids prescribed | 1 (5.9%)  *5 (8.8%)* | 5 (8.5%)  *5 (8.9%)* | 11 (5.9%)  *7 (4.7%)* | 0.752  *0.376* |
| No RA Medication | 0 (0.0%)  *0 (0.0%)* | 0 (0.0%)  *0 (0.0%)* | 1 (0.5%)  *1 (0.7%)* | 1.000  *1.000* |
| Monotherapy |  |  |  |  |
| MTX only | 4 (23.5%)  *9 (15.8%)* | 11 (18.6%)  *8 (14.3%)* | 35 (18.7%)  *32 (21.3%)* | 0.892  *0.480* |
| Other DMARDs only | 0 (0.0%)  *1 (1.8%)* | 5 (8.5%)  *3 (5.4%)* | 15 (8.0%)  *16 (10.7%)* | 0.668  *0.064* |
| Oral Glucocorticoids only | 0 (0.0%)  *0 (0.0%)* | 0 (0.0%)  *2 (3.6%)* | 2 (1.1%)  *0 (0.0%)* | 1.000  *0.045* |
| Parenteral Glucocorticoids only | 2 (11.8%)  *4 (7.0%)* | 3 (5.1%)  *3 (5.4%)* | 10 (5.3%)  *8 (5.3%)* | 0.403  *0.934* |
| Dual therapy |  |  |  |  |
| MTX and other DMARDs only | 4 (23.5%)  *13 (22.8%)* | 9 (15.3%)  *12 (21.4%)* | 40 (21.4%)  *28 (18.7%)* | 0.518  *0.760* |
| MTX and Oral Glucocorticoids only | 0 (0.0%)  *3 (5.3%)* | 3 (5.1%)  *1 (1.8%)* | 3 (1.6%)  *2 (1.3%)* | 0.320  *0.262* |
| MTX and Parenteral Glucocorticoids only | 2 (11.8%)  *9 (15.8%)* | 7 (11.9%)  *6 (10.7%)* | 23 (12.3%)  *17 (11.3%)* | 1.000  *0.641* |
| Other DMARDs and Oral Glucocorticoids only | 1 (5.9%)  *1 (1.8%)* | 0 (0.0%)  *1 (1.8%)* | 1 (0.5%)  *0 (0.0%)* | 0.175  *0.184* |
| Other DMARDs and Parenteral Glucocorticoids only | 0 (0.0%)  *3 (5.3%)* | 5 (8.3%)  *4 (7.1%)* | 18 (9.6%)  *16 (10.7%)* | 0.598  *0.458* |
| Oral Glucocorticoids and Parenteral Glucocorticoids only | 0 (0.0%)  *1 (1.8%)* | 1 (1.7%)  *0 (0.0%)* | 0 (0.0%)  *0 (0.0%)* | 0.289  *0.430* |
| Triple therapy |  |  |  |  |
| MTX, other DMARDs and Oral Glucocorticoids | 0 (0.0%)  *0 (0.0%)* | 1 (1.7%)  *1 (1.8%)* | 4 (2.1%)  *4 (2.7%)* | 1.000  *0.711* |
| MTX, other DMARDs and Parenteral Glucocorticoids | 4 (23.5%)  *13 (22.8%)* | 14 (23.7%)  *15 (26.8%)* | 35 (18.7%)  *25 (16.7%)* | 0.644  *0.220* |
| Medication pyramid |  |  |  |  |
| No RA medication or MTX only or other DMARDs only | 4 (23.5%)  *10 (17.5%)* | 16 (27.1%)  *11 (19.6%)* | 50 (26.7%)  *49 (32.7%)* | 1.000  *0.039* |
| MTX and other DMARDs without Glucocorticoids | 4 (23.5%)  *13 (22.8%)* | 9 (15.3%)  *12 (21.4%)* | 40 (21.4%)  *28 (18.7%)* | 0.518  *0760* |
| Use of Glucocorticoids with or without any other RA medication | 9 (52.9%)  *34 (59.6%)* | 34 (57.6%)  *33 (58.9%)* | 97 (51.9%)  *73 (48.7%)* | 0.736  *0.236* |

Frequency (percentage) reported with p-values based on Fisher’s Exact tests.
 IR – Inadequate Responder Group; HBAR – Higher Baseline Activity Responder Group;
LBAR – Lower Baseline Activity Responder Group
